# Characterization of backbone dynamics using solution NMR spectroscopy to discern the functional plasticity of structurally analogous proteins

**DOI:** 10.1016/j.xpro.2021.100919

**Published:** 2021-10-29

**Authors:** Ashish A. Kawale, Björn M. Burmann

**Affiliations:** 1 Wallenberg Centre for Molecular and Translational Medicine, University of Gothenburg, 405 30 Gothenburg, Sweden; 2 Department of Chemistry and Molecular Biology, University of Gothenburg, 405 30 Gothenburg, Sweden

**Keywords:** Biophysics, Protein Biochemistry, Structural Biology, NMR

## Abstract

The comprehensive delineation of inherent dynamic motions embedded in proteins, which can be crucial for their functional repertoire, is often essential yet remains poorly understood in the majority of cases. In this protocol, we outline detailed descriptions of the necessary steps for employing solution NMR spectroscopy for the in-depth amino acid level understanding of backbone dynamics of proteins. We describe the application of the protocol on the structurally analogous Tudor domains with disparate functionalities as a model system.

For complete details on the use and execution of this protocol, please refer to Kawale and Burmann (2021).

## Highlights

• Backbone dynamics characterization of Tudor domains by solution NMR spectroscopy
• Step-by-step setup and analysis scheme for ^15^N NMR relaxation experiments
• Practical guide for discerning dynamics of structurally analogous proteins
• Protocol applicable for small-to-medium sized proteins and protein-ligand complexes

## Graphical abstract

**Figure:**
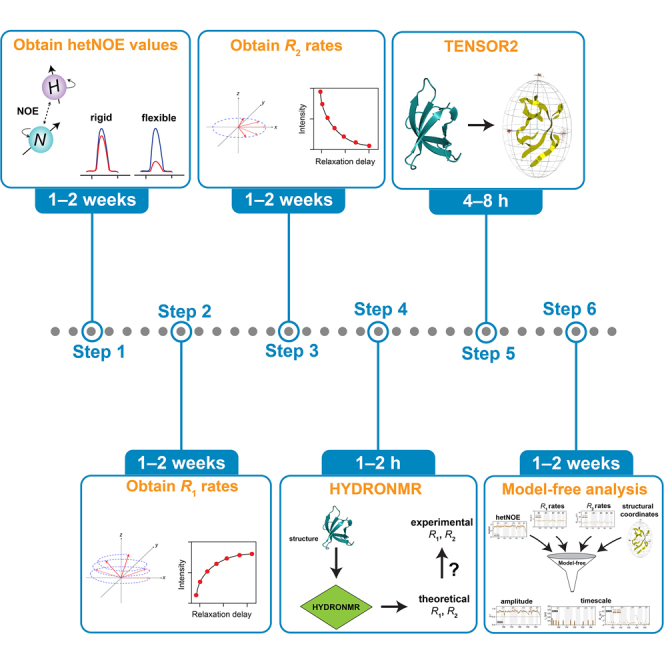

## Before you begin

Proteins are intrinsically dynamic biomolecules. Although a determination of protein structures provides an excellent *ab-initio* information about the structural arrangements of proteins and thereby provides also insight into its function, it predominantly represents merely the time-averaged conformation out of several dynamic conformations adopted by the protein molecule critical for its functionality (Schiro et al., 2020). Therefore, a comprehensive depiction of biomolecular dynamics in solution is of high importance not only to complement the structural information but also for the thorough understanding of the biomolecular functional spectrum (Sekhar and Kay, 2019).

**Figure 1:**
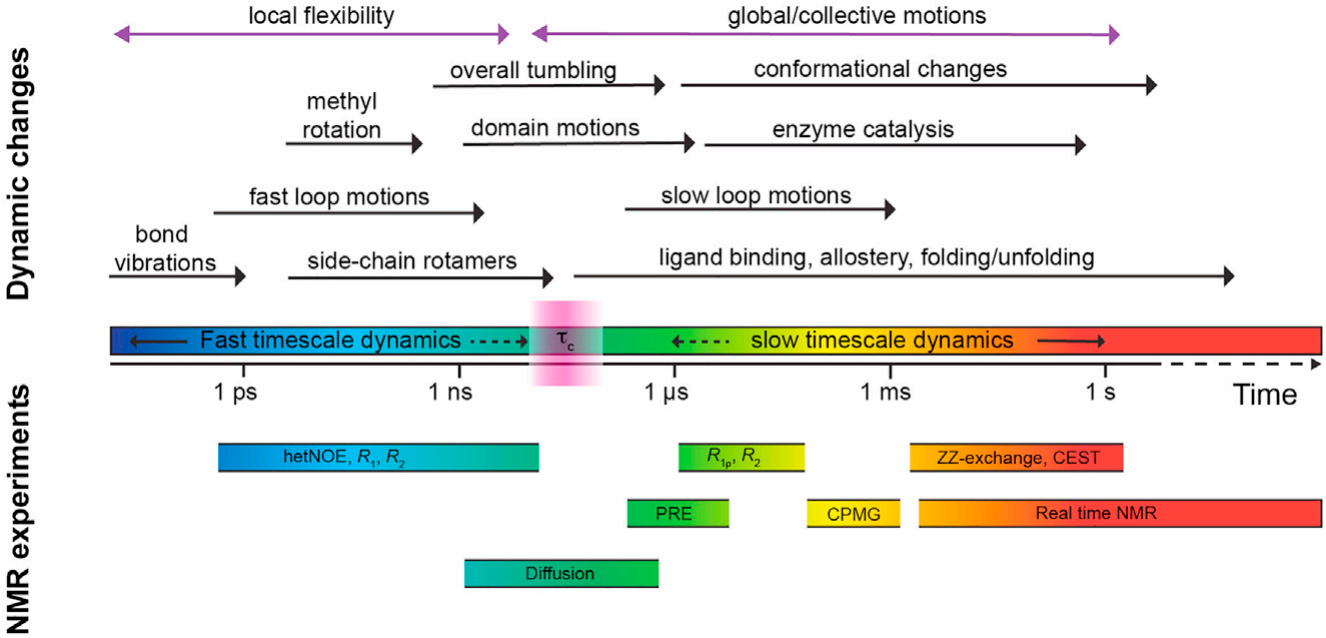
NMR methods to discern protein dynamics at different timescales Overview of the protein dynamics, timescale and available NMR methods to discern atomic-resolution information for the thorough understanding of the biomolecular dynamics. PRE: paramagnetic relaxation enhancement, CPMG: Carr-Purcell-Meiboom-Gill relaxation dispersion; CEST: Chemical exchange saturation transfer.

The protein molecule undergoes a variety of structural transitions at timescales such as the fast (ps–ns) as well as the slow (μs–s) timescale displaying physical motions ranging from bond vibrations, loop motions as well as backbone torsion angle and side-chain rotations with implications in enzyme catalysis, conformational changes, ligand binding and protein folding/unfolding etc. (Figure 1) (Kovermann et al., 2016; Marsh and Teichmann, 2015). Probing protein dynamics involves the systematic characterization of these embedded complex motions occurring at various timescales governed by the conformations accompanied by distinct energy barriers (Kovermann et al., 2016; Markwick et al., 2008). Solution NMR spectroscopy is one of the most powerful as well as most versatile techniques commonly used for the structural and dynamical characterization of biomolecules. The ^15^N NMR relaxation experiments for the characterization of protein backbone dynamics are based on the 2D [^15^N,^1^H] heteronuclear single quantum coherence (HSQC) experiment which provides information about all ^15^N atoms bound to ^1^H atoms (Bodenhausen and Ruben, 1980). In the case of uniformly ^15^N ([*U*-^15^N] or [*U*-^13^C,^15^N]) labelled proteins this experimental approach yields one backbone amide NH peak for each amino acid residue except proline. Thus, this experiment provides atomic resolution information for each backbone amide moiety of the protein molecule. The ^15^N relaxation experiments described in this protocol rely on the intensity changes of the backbone amide peaks providing site-specific information crucial to deciphering timescales and amplitudes of these dynamic fluctuations with atomic precision (Kleckner and Foster, 2011).

**Figure 2:**
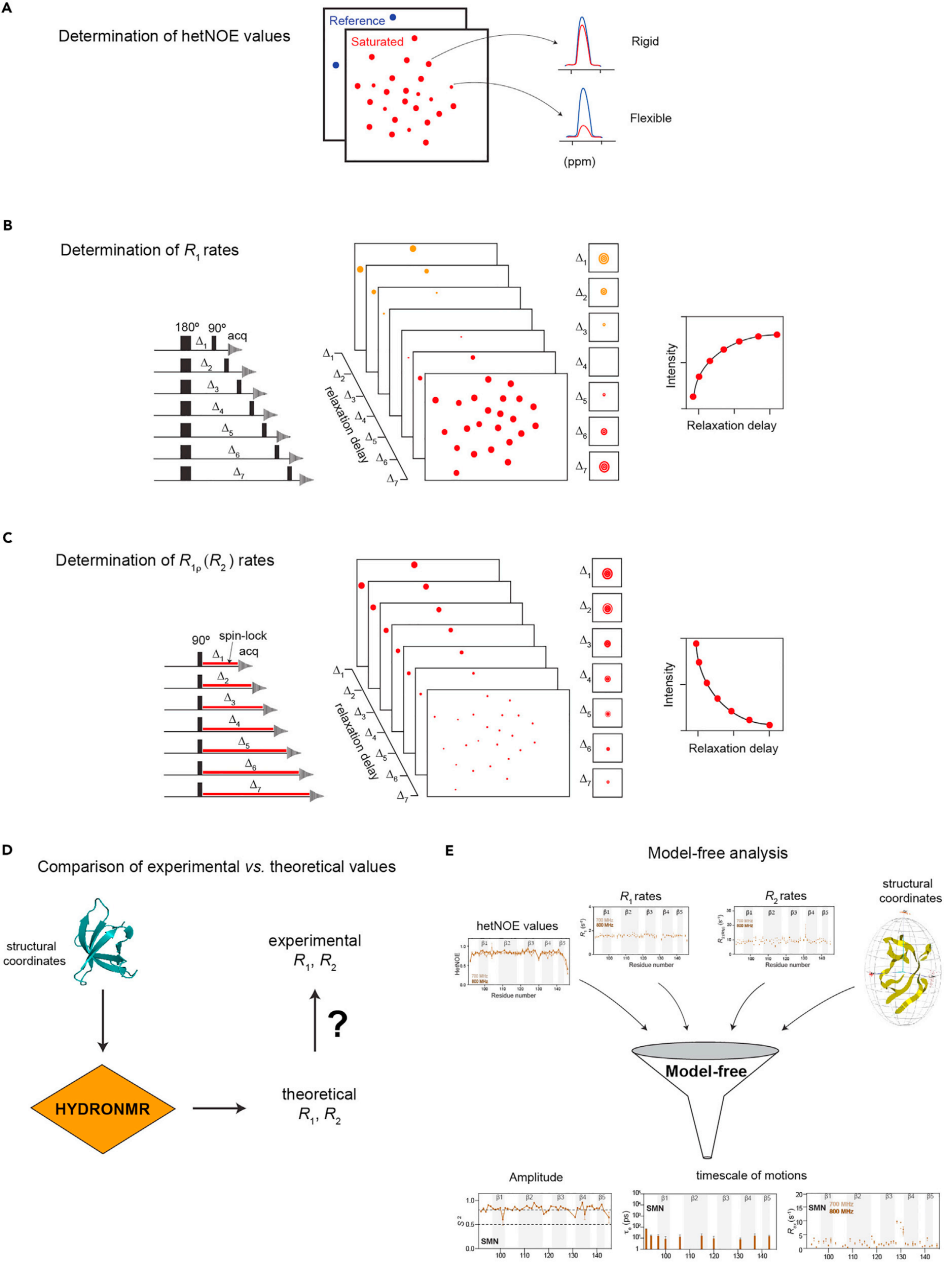
Schematic representation of the steps involved in the backbone dynamics characterization (A) Schematic diagram for the interpretation of hetNOE spectra. (B) T_1_ (inversion recovery) experiment pulse sequence diagram, representative spectra at each delay point and the graph of the peak intensity versus the relaxation delay for each residue. (C) T_1ρ_ experiment pulse sequence diagram, representative spectral changes observed at each delay point and the graph of the peak intensity versus the relaxation delay observed for each residue. (D) Validation using HYDRONMR computed R1, R2 rates. (E) Model-free anaysis step to provide physically meaningful dynamic motional analysis from the experimental relaxation data.

This protocol consists of a step-by-step setup and analysis scheme for the measurements of ^15^N protein NMR backbone relaxation experiments using Tudor domain proteins as a model system (Figure 2). We then describe the data evaluation procedure followed by the description of the steps for the HYDRONMR (Garcia de la Torre et al., 2000) and Tensor2 (Dosset et al., 2000) analysis. Lastly, we outline the procedure for the Model-free analysis (Lipari and Szabo, 1982a, 1982b) using relaxation data obtained at two magnetic field strengths quintessential to draw meaningful conclusions about the dynamical aspects of the protein functions by using the approach developed by d’Auvergne and Gooley applicable *via* the program relaxGUI (Bieri et al., 2011). The main advantage of the outlined protocol is that the data can be collected in aqueous buffers under near-physiological conditions. Although the protocol presented is used for well-folded proteins around ∼8 kDa in size, we also outline experimental adaptations so that this protocol can serve as a template to obtain and interpret relaxation data to discern relaxation properties of the protein NH groups also for medium-sized (up to 25–30 kDa) proteins as well as protein-ligand complexes.

The protocol requires the following things to be set up before beginning. The individual steps are summarized to provide the necessary details for protein production. For a more detailed general descriptions about protein expression refer to the Sambrook protocol (Sambrook and Russell, 2001).

### NMR sample preparation

**Timing: 2–3 weeks**

1. Obtain the following constructs for recombinant production of SMN, NusG and RfaH Tudor domain proteins from *E. coli.*
  a. The SMN Tudor domain construct (82–147), cloned into a pET24d vector with an amino-terminal hexahistidine-GST tag followed by the TEV protease cleavage site (Sprangers et al., 2003b) (a kind gift by M. Sattler, TU Munich).
  b. The NusG Tudor construct (123–181) cloned into a pET28b(+) vector containing an amino-terminal hexahistidine tagged Sumo protein solubility tag (Kawale and Burmann, 2021)(available upon request from the Burmann Lab, Gothenburg).
  c. The RfaH Tudor construct (101–162) cloned into a pET28b(+) vector backbone harboring an amino-terminal hexahistidine tagged Sumo fusion protein (Burmann et al., 2012) (purchased from GenScript; available upon request from the Burmann Lab, Gothenburg).

**CRITICAL:** Inconsistent design of domain boundaries can lead to disparities between protein stability and structure and can cause a mismatch with published NMR data. Perform DNA sequencing to confirm the DNA sequence.

2. Transformation of Tudor domain constructs in chemically competent bacteria.
  a. Take chemical competent *E. coli* BL21 Star^TM^ (λDE3) cells out of −80°C and thaw on ice (∼ 30 min).
  b. Take LB agar plates containing Kanamycin from the fridge and incubate at 37°C in an incubator.
  c. Pipette 1–2 μL of plasmid (∼50 ng/μL) into 25–50 μL competent cells and mix gently.
  d. Incubate the cells on ice for 20–30 min.
  e. Heat shock at 42°C for 45 s on a heating block.
  f. Incubate the cells on ice for 2 min.
  g. Add 400 μL of LB medium and incubate at 37°C for 1 h on a shaking incubator.
  h. Centrifuge the cells at 3400 **×** *g* for 2 min and remove the 400 μL supernatant solution.
  i. Gently mix the cells and streak out evenly on the LB agar plates from the incubator.
  j. Incubate plates for 16–20 h at 37°C.
  k. Seal the agar plates with parafilm and transfer to the fridge (4°C) to prevent the growth of satellite colonies.

**Pause point:** Agar plates can be kept at the fridge (4°C) for about 2 weeks before proceeding to the next step, but for the best results proceed to the next step within a week.

**CRITICAL:** Maintain a sterile environment while working with bacterial cultures. Clean all the equipment with 70% Ethanol and work under a flame or Laminar airflow to prevent contamination.

3. Expression of [*U*-^15^N]-labelled Tudor domain proteins.
  a. Pick a single colony from the transformed plate by using a sterile loop to inoculate the 5 mL LB medium containing Kanamycin (50 μg/mL final concentration) and incubate at 37°C, 200 rpm for over-day i.e., 6–8 h.
  b. Inoculate 50 mL of M9 medium in 250 mL Erlenmeyer flask with 1 mL of over-day grown LB preculture and incubate at 37°C, 200 rpm for 16–20 h.
  c. Transfer the M9 preculture to the main M9 culture (1 L) in a 5 L Erlenmeyer flask and grow at 37°C, 200 rpm. Monitor the optical density (OD_600 nm_) using UV/Vis-spectrophotometer until it reaches 0.6–0.8.
  d. Add 0.5 mM Isopropyl β-D-thiogalactoside (IPTG) for the induction of the RfaH Tudor domain and 1 mM IPTG for the induction of SMN and RfaH Tudor protein.
  e. Incubate cells at 25°C, 200 rpm for 16–20 h for NusG and RfaH Tudor domain constructs whereas for the SMN Tudor domain culture incubate cells for 4 h.
  f. Harvest bacterial cultures by centrifugation at 4000 **×** *g* for 20 min at 4°C.
  g. Discard the supernatant and retain the cell pellets.

**Pause point:** Store cell pellets at −80°C until further use. Pellets can be stored at −80°C for up to 1–2 months. Prior to proceeding to the next step, thaw them at 20°C–22°C and later keep on them ice.

4. Purification of [*U*-^15^N]-labelled Tudor domain proteins.
  a. Add one cOmplete, EDTA-free Protease Inhibitor Cocktail tablet (Roche), 5 μL HL-SAN DNase I (ArticZymes) and 10 mM MgSO_4_ to the lysis buffer (50 mL buffer per 10 g of wet cell-pellet weight).
  b. Use the above-mentioned buffer solution to resuspend the resultant cell pellets harvested after centrifugation by gently pipetting up and down until a homogenous solution is achieved.
  c. Turn on the Emulsiflex C3 (Avestin) homogenizer and keep it at 4°C.
  d. Pass the resuspended cell mass at least three times through an Emulsiflex C3 (Avestin) homogenizer at 4°C to achieve a clear cell lysate indicative of near-complete cell lysis.
  e. Centrifuge at 19000 **×** *g* for 45 min at 4°C to separate the cell debris from the lysate.
  f. Transfer the clear supernatant into 50 mL tubes and keep it on ice and discard the pellet containing debris.
  g. Equilibrate a manually packed Ni^2+^–NTA (HisPur^TM^ resin, Thermo Fisher Scientific) gravity flow column with lysis buffer and load the clear supernatant at least twice onto it at 20°C–22°C. Collect the flow-through for SDS-PAGE analysis.
  h. Wash the column by passing 10 column volumes (CV) of lysis buffer and collect the flow-through for SDS-PAGE analysis.
  i. Perform an additional washing step by passing lysis buffer supplemented with 25 mM Imidazole to remove non-specifically bound proteins.
  j. Pass 5 CV elution buffer for the elution of bound proteins and collect flow-through in fresh 50 mL conical tubes and place them on ice.
  k. Confirm the presence and degree of purification of the desired proteins by running an SDS-PAGE.
  l. Add human Sumo protease/TEV protease (His-tagged SenP1: Addgene #16356 (Mikolajczyk et al., 2007); His-tagged TEV-protease: Addgene #8827 (Kapust et al., 2001)) and put the proteins for dialysis for 16–20 h in a dialysis buffer for the subsequent Sumo-tag/GST-tag cleavage.
  m. Confirm the Sumo-tag/GST-tag cleavage by running an SDS-PAGE.
  n. If the cleavage is not complete, add additional protease and incubate at RT for 2–3 h.
  o. Perform a second Ni^2+^–NTA gravity column purification step to remove the cleaved Sumo-tag/GST- tag or SenP1/TEV protease.
  p. Retain the flow-through fractions containing the protein of interest for subsequent purification, keep on ice.
  q. Perform further washing and elution steps with the respective buffers for the SDS-PAGE analysis together with the collected flow-through fractions.
  r. Run SDS-PAGE to confirm the purification process and the presence of desired protein band.
  s. Combine all the fractions containing the protein of interest and concentrate up to 1–2 mL using Vivaspin 15R centrifugal concentrators (Sartorius) with 3 kDa MWCO (Molecular weight cut-off) at 4°C.
  t. Equilibrate a HiLoad 16/600 Superdex75 prep grade column (GE Healthcare) with size-exclusion buffer on an ÄKTA FPLC (GE Healthcare) at 4°C for the size exclusion chromatography and subsequently apply the concentrated protein fractions for the final purification step.
  u. Monitor UV absorbance to identify the protein of interest and confirm the presence and purity by SDS-PAGE.
  v. Combine all the fractions containing the protein of interest and buffer exchange to the respective NMR buffers using Vivaspin 15R centrifugal concentrators (Sartorius) with 3 kDa MWCO (Molecular weight cut-off).

**Pause point:** Flash-freeze protein aliquots with the help of liquid nitrogen, and store at −80°C until further use for several months. Before proceeding to the next step, thaw the proteins at 20°C–22°C.

**CRITICAL:** Always store buffers in the fridge or the cold room. It is recommended to use fresh Ni^2+^–NTA resin for each protein purification to avoid contamination. Use clean chromatographic columns and regularly clean all parts of the ÄKTA system for obtaining the best results. Be careful not to pass air into the size exclusion column. Monitor the DNA contamination by checking the ratio of the absorbance at 260 and 280 nm (ratio of 0.5–0.6 reflects pure protein) using a nanodrop or alternate a UV/VIS spectrophotometer.

### Sample quality check and BMRB assignment transfer

**Timing: 1–2 days**

5. Quality check by 1D (&/or 2D) NMR spectroscopy
  a. Record ^1^H 1D NMR spectrum for each Tudor domain in the respective buffer condition at increasing sample concentrations (typically in the range of 0.1–0.5 mM).
  b. Process and analyze the 1D spectra using TopSpin software.
  c. Monitor the consistency in the protein amide region of the ^1^H 1D spectra to find out if concentration-dependent aggregation effects occur.
  d. Select the highest sample concentration (≤ 0.5 mM) for the backbone data measurement, where the protein ^1^H 1D spectra is consistent with the lower protein concentrations.

***Note:*** 2D NMR spectroscopy can also be applied to perform the above-mentioned procedure, though it is more time-consuming than 1D NMR spectroscopy.

**CRITICAL:** Always use clean and dry high-quality sample tubes. Wipe the sample tube using clean tissue paper. Use the correct spinner type, which can hold the sample tightly. Adjust the sample depth using a sample depth gauge and turn on lift air prior to inserting the sample into the NMR instrument. Ensure that the NMR spectrometer is set to the correct temperature before inserting the sample.

6. BMRB assignment transfer
  a. Record [^15^N,^1^H] heteronuclear single quantum coherence (HSQC) spectra for each Tudor domain in the respective buffer conditions.
  b. Use TopSpin4 (Bruker BioSpin) to process and analyze the spectra.
  c. Download the sequence-specific resonance assignment for each Tudor domain from the Biological Magnetic Resonance Data Bank website (https://bmrb.io) in the NMR_STAR v3 file format. The respective BMRB Entry codes are 15490 (NusG Tudor), 17615 (RfaH Tudor), 18005 (SMN-Tudor) (Burmann et al., 2012; Kawale and Burmann, 2020; Mooney et al., 2009; Tripsianes et al., 2011).
  d. Open the spectrum in NMRFAM-sparky (Lee et al., 2015). More information regarding sparky commands and usage of the program can be found under (https://nmrfam.wisc.edu/nmrfam-sparky-distribution) and (https://www.cgl.ucsf.edu/home/sparky/manual/indx.html).
  e. From the NMRFAM-SPARKY *Menubar* go to the NMRFAM, click on *Utilities*, then to the *NMRSTAR 3.1 to SPARKY* option or alternatively type ***ns*** in the NMRFAM-sparky command line.
  f. Select the downloaded NMRstar file and type the file name to convert it to the required sparky resonance format (.list)
  g. Type ***rl*** to open the resonance list and click on the *Load* option at the bottom and select the recently converted resonance .list file to load the sequence-specific resonance assignments of the protein.
  h. From the main NMRFAM-SPARKY Menu bar go to the *NMRFAM* option, click on the *Superfast* assignments, then to the Transfer and simulate assignments option or alternatively type ***ta*** in the NMRFAM-sparky command line.
  i. Select *N-HSQC* as the type of the spectrum and click on the transfer option, which will transfer the BMRB downloaded assignments onto the [^15^N,^1^H] HSQC spectrum.
  j. Perform the outlined steps above for all the Tudor domains datasets.

**CRITICAL:** In case of a mismatch between the BMRB assignments and your spectrum peaks, perform chemical shift referencing by using the DSS (sodium trimethylsilylpropanesulfonate) standard. The following website pages describe a quick guide for the process.

http://www.iiserpune.ac.in/∼cjeet/wordpress/2008/07/14/chemical-shift-referencing-calculator/ and

https://lsom.uthscsa.edu/biochemistry/core-facilities/biomolecular-nmr-core/technical-resources/chemical-shift-referencing-and-temperature-calibration/

## Key resources table

**Table:** 

REAGENT or RESOURCE	SOURCE	IDENTIFIER
**Bacterial and virus strains**		

*E. coli* BL21 Star^TM^ (λDE3)	Thermo Fisher Scientific	Cat. #C601003

**Chemicals, peptides, and recombinant proteins**		

KH_2_PO_4_	VWR	Cat. #26936.293
K_2_HPO_4_	Merck	Cat. #P3786
Na_2_HPO_4_	Sigma-Aldrich	Cat. #S9763
Glucose	VWR	Cat. #101176K
FeCl_2_∗4H_2_O	Sigma-Aldrich	Cat. #103861
CaCl_2_∗2H_2_O	Merck	Cat. #102382
H_3_BO_3_	Sigma-Aldrich	Cat. #B-6768
CoCl_2_∗6H_2_O	Sigma-Aldrich	Cat. #C8661
CuCl_2_∗2H_2_O	Sigma-Aldrich	Cat. #22178-3
ZnCl_2_	VWR	Cat. #29157.234
Na_2_MoO_4_∗2H_2_O	ACROS ORGANICS	Cat. #206371000
MnCl_2_∗4H_2_O	Sigma-Aldrich	Cat. #105927
D-Biotin	Sigma-Aldrich	Cat. #B4501
Choline chloride	Sigma-Aldrich	Cat. #C7017
Folic acid	Sigma-Aldrich	Cat. #F7876
Myo-Inositiol	Sigma-Aldrich	Cat. #I5125
Nicotinamide	Sigma-Aldrich	Cat. #72340
D-pantothenic acid ½ Ca	Sigma-Aldrich	Cat. #21210
Pyridoxal-HCl	Sigma-Aldrich	Cat. #93759
Riboflavin	Sigma-Aldrich	Cat. #R4500
Thiamine HCl	Sigma-Aldrich	Cat. #T4625
KCl	Merck	Cat. #P3911
NaCl	VWR	Cat. #27800.360
MgSO_4_	Merck	Cat. #230391
Kanamycin Sulfate	Applichem GmbH	Cat. #A4789,0025
(^15^NH_4_)Cl	Merck	Cat. #299251
Isopropyl β-D-thiogalactoside (IPTG)	Fisher Scientific	Cat. #10021793
cOmplete, EDTA-free Protease Inhibitor Cocktail tablet	Roche	Cat. #5056489001
HL-SAN DNase I	ArticZymes	Cat. #70910-202
HisPur^Tm^ resin	Thermo Fisher Scientific	Cat. #88223
Imidazole	Alfa Aesar	Cat. #47274.0E
Tris(hydroxymethyl)aminomethane	VWR	Cat. #103157P
HEPES	VWR	Cat. #441487M
DTT	Sigma-Aldrich	Cat. #D0632
D_2_O	Merck	Cat. #151882
Tryptone	VWR	Cat. # 97063-388
Yeast extract	VWR	Cat. #97064-370

**Deposited data**		

SMN Tudor	(Sprangers et al., 2003a)	BMRB: 18005
NusG Tudor	(Mooney et al., 2009)	BMRB: 15490
RfaH Tudor	(Burmann et al., 2012)	BMRB: 17615
SMN Tudor	(Sprangers et al., 2003a)	PDB: 1MHN
NusG Tudor	(Mooney et al., 2009)	PDB: 2JVV
RfaH Tudor	(Burmann et al., 2012)	PDB: 2LCL

**Recombinant DNA**		

Plasmid: pET24b_SMN_Tudor	(Sprangers et al., 2003b)	N/A
Plasmid: pET28b_Sumo_NusG_Tudor	(Kawale and Burmann, 2021)	N/A
Plasmid: pET28b_Sumo_RfaH_Tudor	(Kawale and Burmann, 2021)	GenScript
Plasmid: His-tagged TEV-protease	(Kapust et al., 2001)	Addgene: Cat. #8827
Plasmid: His-tagged SenP1	(Mikolajczyk et al., 2007)	Addgene: Cat. #16356

**Software and algorithms**		

TopSpin4.0.4	Bruker BioSpin	https://www.bruker.com/en/products-and-solutions/mr/nmr-software/topspin.html
NMRFAM-sparky	(Lee et al., 2015)	https://nmrfam.wisc.edu/nmrfam-sparky-distribution
relaxGUI	(Bieri et al., 2011)	https://www.nmr-relax.com
HYDRONMR	(Garcia de la Torre et al., 2000)	http://leonardo.inf.um.es/macromol/programs/hydronmr/hydronmr.htm
Tensor2	(Dosset et al., 2000)	https://www.ibs.fr/research/scientific-output/software/tensor/?lang=fr
NMRBox	(Maciejewski et al., 2017)	https://nmrbox.org
Microsoft Excel (or similar spreadsheet application for the calculations)	Microsoft	https://www.microsoft.com/en-us/microsoft-365/excel
GraphPrism (or similar data analysis software)	GraphPad	https://www.graphpad.com/scientific-software/prism/
NMRPipe/NMRDraw (optional)	(Delaglio et al., 1995)	https://www.ibbr.umd.edu/nmrpipe/index.html
PyMol (optional)	Schrödinger	https://pymol.org/2/
FAST ModelFree (optional)	(Cole and Loria, 2003)	http://ursula.chem.yale.edu/∼lorialab/software.php

**Other**		

Vivaspin 3 (MWCO) centrifugal concentrators	Sartorius	Cat. #VS0692
HiLoad 16/600 Superdex75 prep grade column	GE Healthcare	Cat. #28989333
5 mm NMR tubes	ARMAR AG, Switzerland	Cat. #032100.5045

## Materials and equipment

The protocol requires two high magnetic field strength NMR instruments (e.g., Bruker BioSpin; 500–1,200 MHz ^1^H frequency) running respective data acquisition software (e.g., TopSpin, Bruker BioSpin).

**CRITICAL:** Depending on the availability of high-field NMR spectrometers, a larger spread of the magnetic field strength is advantageous, e.g. 500 and 900 MHz.

***Optional:*** NMR instruments equipped with cryogenic probes (e.g. Prodigy- or TCI-probes, Bruker BioSpin) for increased sensitivity.

***Note:*** This protocol makes use of the NMR instruments, accessories, and associated software from Bruker BioSpin, but it can be easily adapted to any other NMR platforms (e.g. Jeol or Agilent/Varian).

As temperature affects the kinetic properties of the protein samples; constant temperature needs to be set during all the experiments and continuous checking ensures stability of the analysis of the dynamic properties.

High-resolution structures and backbone NMR assignments of the proteins under study are required. It is essential to use uniformly ^15^N ([*U*-^15^N] or [*U*-^13^C,^15^N]) labelled protein samples (Tudor domain) with a >95% purity in the respective buffer solutions for the NMR data measurements. In addition, 10% D_2_O needs to be supplemented to the samples for the NMR lock signal. The protein sample concentrations of ≥ 0.1 mM with sample volumes ranging between 180–500 μL are required depending on the type of NMR tube used.

**CRITICAL:** Choose an appropriate protein concentration. For Tudor domains, no aggregation/oligomerization was observed for samples ∼500 μM, whereas for larger proteins often the concentration has to be reduced to 100–200 μM to avoid these effects (See before you begin, 5. Quality check by 1D (&/or 2D) NMR spectroscopy).

The media and buffer recipes used in this protocol are listed below.

**CRITICAL:** Read the Material Safety Data Sheet (MSDS), pay attention to the safety concerns and handle any hazardous chemicals with care while preparing the following recipes.

**Table:** LB media (1 L)

Reagent	Final concentration	Amount
Tryptone	140.7 mM	10 g
NaCl	171.1 mM	10 g
Yeast Extract	15.7 mM	5 g
Water	n/a	950 mL

To prepare 1 L LB media, weigh and mix the above reagents in 950 mL Milli-Q water. Mix the reagents until the solutes have dissolved completely. Adjust final volume to 1 L using Milli-Q water. Autoclave to sterilize the final medium. The media can be stored at 25°C for up to 1 week.

**Table:** M9 minimal media

Reagent	Final concentration	Amount
Na_2_HPO_4_	33.7 mM	6 g
KH_2_PO_4_	22 mM	3 g
NaCl	8.55 mM	0.5 g
Solution Q∗	n/a	1mL
MgSO_4_ powder	2 mM	240.7 mg
Vitamins mix∗∗	n/a	15 mL
Glucose	11.1 mM	2 g
[^15^N] NH_4_Cl	18..4 mM	1 g
Kanamycin	50 μg/mL	50 mg/mL
Water (autoclaved)	n/a	900 mL

M9 minimal media (Sambrook and Russell, 2001) is used to generate uniformly ^15^N-labelled [*U*-^15^N] proteins. To prepare 1L M9 minimal media, weigh and dissolve the above reagents in 900 mL autoclaved Milli-Q water. Use Milli-Q water to adjust the final volume to 1 L. Sterilize by filtration using a 0.2 μm filter. The media can be stored at 25°C for up to 1 week.

**Table:** ∗Solution Q (1000**×** stock)

Reagent	Final concentration	Amount
FeCl_2_∗4H_2_O	25.2 mM	5 g
CaCl_2_∗2H_2_O	1.3 mM	184 mg
H_3_BO_3_	1 mM	64 mg
CoCl_2_∗6H_2_O	0.1 mM	18 mg
CuCl_2_∗2H_2_O	0.02 mM	4 mg
ZnCl_2_	2.5 mM	340 mg
Na_2_MoO_4_∗2H_2_O	2.9 mM	605 mg
MnCl_2_∗4H_2_O	0.2 mM	40 mg

To prepare 1 L Solution Q, weigh and dissolve the above reagents in 900 mL autoclaved

Milli-Q water. Sterilize by filtering the solution by a 0.2 μm filter and store at 4°C. The solution can be stored at 4°C for up to several months.

**Table:** ∗∗Vitamins mix (100**×**)

Component	Final concentration	Amount
D-Biotin	0.4 mM	0.1 g
Choline chloride	0.7 mM	0.1 g
Folic acid	0.2 mM	0.1 g
Myo-Inositiol	1.1 mM	0.2 g
Nicotinamide	0.8 mM	0.1 g
D-pantothenic acid ½ Ca	0.2 mM	0.1 g
Pyridoxal-HCl	0.5 mM	0.1 g
Riboflavin	0.03 mM	0.01 g
Thiamine HCl	0.3 mM	0.1 g
NaCl	145.4 mM	8.5 g

To prepare 1 L Vitamins mix solution, dissolve the above reagents in 700 mL Milli-Q water. Adjust pH between 6.8 to 7.2 at 25°C. Adjust final volume to 1 L using Milli-Q water. Filter using a 0.2 μm filter. Aliquot to 10 mL fractions. Store at −20°C. The solution can be stored at −20°C for up to several months.

Lysis/Load buffer

**Table:** 50 mM Tris pH 8.0, 500 mM NaCl, 5 mM Imidazole

Reagent	Final concentration	Amount
Tris-base	50 mM	6.06 g
NaCl	500 mM	29.22 g
Imiazole	5 mM	0.34 g

To prepare 1 L Lysis/Load buffer, dissolve the above reagents in 700 mL Milli-Q water. Adjust pH of the buffer to 8.0 at 25°C. Adjust final volume to 1 L using Milli-Q water. Filter using a 0.2 μm filter. The buffer can be stored at 4°C for 1–2 weeks.

Elution buffer

**Table:** 50 mM Tris pH 8.0, 500 mM NaCl, 250 mM Imidazole

Reagent	Final concentration	Amount
Tris-base	50 mM	6.06 g
NaCl	500 mM	29.22 g
Imidazole	250 mM	17.02 g

To prepare 1 L Elution buffer, dissolve the above reagents in 700 mL Milli-Q water. Adjust pH of the buffer to 8.0 at 25°C. Adjust final volume to 1 L using Milli-Q water. Filter using a 0.2 μm filter. The buffer can be stored at 4°C for 1–2 weeks.

Dialysis buffer

**Table:** 50 mM Tris pH 8.0, 150 mM NaCl, 1 mM DTT

Reagent	Final concentration	Amount
Tris-base	50 mM	30.30 g
NaCl	150 mM	43.85 g
DTT	1 mM	0.75 g

To prepare 5 L Lysis/Load buffer, dissolve the above reagents in 4000 mL Milli-Q water. Adjust pH of the buffer to 8.0 at 25°C. Adjust final volume to 5 L using Milli-Q water. The buffer can be stored at 4°C for 1–2 weeks.

Size-exclusion buffer

**Table:** 20 mM Tris pH 8.0, 150 mM NaCl, 1 mM DTT

Reagent	Final concentration	Amount
Tris-base	20 mM	2.42 g
NaCl	150 mM	8.77 g
DTT	1 mM	0.15 g

To prepare 1 L Size exclusion buffer, dissolve the above reagents in 700 mL Milli-Q water. Adjust pH of the buffer to 8.0 at 25°C. Adjust final volume to 1 L using Milli-Q water. Filter the buffer using a 0.2 μm filter. The buffer can be stored at 4°C for 1–2 weeks.

NusG/RfaH Tudor NMR Buffer

**Table:** 25 mM HEPES pH 7.5, 50 mM NaCl

Reagent	Final concentration	Amount
HEPES	25 mM	0.60 g
NaCl	50 mM	0.29 g

To prepare 100 mL NusG/RfaH NMR Buffer, dissolve the above reagents in 70 mL Milli-Q water. Adjust pH of the buffer to 7.5 at 25°C. Adjust final volume to 100 mL using Milli-Q water. Filter using a 0.2 μm filter. The buffer can be stored at 4°C for 1–2 weeks.

SMN Tudor NMR Buffer

**Table:** 25 mM potassium phosphate pH 6.8, 20 mM NaCl, 2 mM DTT

Reagent	Final concentration	Amount
K_2_HPO_4_	11.4 mM	0.20 g
KH_2_PO_4_	13.6 mM	0.18 g
NaCl	20 mM	0.12 g
DTT	2 mM	0.03 g

To prepare 100 mL SMN Tudor NMR Buffer, dissolve the above reagents in 70 mL Milli-Q water. Adjust pH of the buffer to 6.8 at 25°C. Adjust final volume to 100 mL using Milli-Q water. Filter using a 0.2 μm filter. The buffer can be stored at 4°C for 1–2 weeks.

## Step-by-step method details

The protocol involves six major steps which are repeated on each protein structure under consideration (Figure 2).

### Step 1: Determination of hetNOE values at two magnetic fields

**Timing: 2 weeks**

This step illustrates the procedure for the determination of hetNOE values by measuring ^15^N{^1^H}-NOE experiment (Renner et al., 2002) (Figure 2A). This experiment incorporates through-space magnetization transfer *via* the dipolar coupling from the ^1^H to the directly attached ^15^N nucleus providing information regarding the motions of the individual N-H Bond vectors of the protein molecules. A considerable decrease in the NOE intensity value in comparison to the average NOE intensity is observed for the residues undergoing fast timescale motions (ps–ns timescale). The ^15^N{^1^H}-NOE experiment typically employs measurements of two different spectra with (termed as *Saturated*) and without (termed as *Reference*) proton saturation in an interleaved fashion. The ratio of the intensities in the *Reference* and *Saturated* spectra corresponds to the value of the steady-state heteronuclear NOEs for each residue.

1. Measurement on the NMR instrument with magnetic field strength B_1_ (for e.g., 11.7 Tesla (T) with a ^1^H-frequency of 500 MHz).
  a. Data acquisition
    i. Create a new dataset and experiment.
    ii. Load the Bruker pulse sequence for the 2D ^15^N{^1^H}-NOE experiment (e.g., *hsqcnoef3gpsi* from the standard Bruker pulse sequence library).
    iii. Adjust the sample temperature on the NMR instrument to the desired value based on the stability of the sample and the quality of the spectrum.
    iv. Insert the sample into the magnet.
    v. Perform steps for the lock, tune and match and shim.
    vi. Calibrate the ^1^H pulse length (90° hard pulse) for the sample using a standard calibration experiment.
    vii. Calibrate the water position to determine the ^1^H radio frequency carrier position.
    viii. Calibrate the ^15^N pulse length (90° hard pulse) for the sample.
    ix. Adjust the desired ^15^N radio frequency carrier length from the previous experiments (typically in the range of 116–122) and the sweep width of the spectrum to observe all resonances. If you have no idea from previous experiments, use 119 ppm as a carrier position and 36 ppm as a sweep width.
      ***Optional:*** Calibrate the ^13^C pulse length (90° hard pulse) for the sample if using an [*U*-^13^C,^15^N] labeled sample and adjust the desired ^13^C radio frequency carrier to 110 ppm. This approach ensures that the ^15^N is decoupled from neighboring Cα and CO resonances.
    x. Adjust the sweep widths for ^1^H and ^15^N and acquisition times.
    xi. Set 2048 × 256 complex data points.
    xii. Set the number of scans (**ns**) to the desired value, which is the compromise between obtained signal to noise and the availability of measurement time. In the example spectra, **ns** of 8 was used for 500 μM sample concentration. Increase the number of scans value, using the **ns** parameter for dilute samples accordingly.
    xiii. Adjust the recycling delay, **d1**, to at least 3s. It is important to have a longer *d1* delay value to improve the accuracy of the measurement (Renner et al., 2002).
    xiv. Check the receiver gain by typing **rga** in the topspin command line.
    xv. Type **zg** to start the acquisition.
      **CRITICAL:** Check the pulse sequence for the phase cycling value and determine the *ns* value accordingly to allow the selection of the proper coherence pathway. Typically, it is a multiple of 4, 8, or 16.
  b. Data processing
    i. After the experiment is finished, type **split** in the Topspin command line to separate the interleaved *Reference* and *Saturated* spectra.
    ii. Enter **2** as the rows to split and desired experiment number to store the new dataset, which will create two new separate datasets. The first will be the *Reference* and the second will be the *Saturated* experiment dataset.
    iii. First process the *Reference* spectrum by using standard processing parameters, phase correction values and the 2D Fourier transformation command **xfb.**
    iv. Process the *Saturated* experiment using the same processing parameters as the reference spectrum. Most importantly the intensity scaling factor needs to be the same. To check the intensity scaling factor of the *Reference* spectrum, open the reference spectrum and type **nc_proc.** Note this value and open the *Saturated* experiment and type in the command line **xfb nc_proc x**, where x is the intensity scaling factor for the reference spectrum in an integer value.
    v. Convert Bruker files into the .ucsf file using the *bruk2ucsf* macro by NMRFAM-SPARKY.
    vi. Open .ucsf files for both *Reference* and *Saturated* spectra in NMRFAM-SPARKY. Transfer resonance assignments from the BMRB (see before you begin, 6. BMRB assignment transfer).
    vii. To save the project file, click on the *File* option from the NMRFAM-Sparky main menu bar followed by *Project* and *Save As…* and provide a suitable file name for the project.
    viii. Type **lt** to open the peak list of the reference spectrum. Click on the *options* and check the *Data height* box. Click *Apply* and then *Close.* Now you can see the additional column displaying data height for each peak.
    ix. Click *save* to save the list for the reference spectrum.
    x. For the noise estimation. Type **st** in the command line and note the value for the estimated noise.
    xi. Perform the above-mentioned steps for the processing of the *Saturated* spectrum.
  c. Data analysis using relaxGUI
    i. Open relaxGUI. The detailed information regarding the use of relaxGUI can be found at (https://www.nmr-relax.com/manual/The_GUI.html).
    ii. Start new *Steady-state NOE analysis* by clicking on *File*, *New analysis*, *Steady-state NOE.*
    iii. Enter NMR frequency and define the Results directory path.
    iv. Load N and H spins by clicking on the Spin editor function by using a PDB structure file or file containing sequence data.
    v. In the main relax window, click on the *Add* button under the Spectra list option to open the Peak intensity loading wizard.
    vi. Next step involves specifying the data file names by selecting correct file paths for both *Reference* and *Saturated* peak lists exported from the NMRFAM-Sparky followed by specifying the spectrum ID Strings (1, 2 etc.). Click *Apply* and *Next.*
    vii. Choose *Baseplane RMSD* for the analysis of the peak intensity error and in the next step provide noise associated with each *Reference* and *Saturated* spectrum from NMRFAM-SPARKY as outlined in 1b. x.
    viii. In the next step specify the spectrum type (i.e., *Reference* or *Saturated*) to the correct spectrum ID string (1, 2 etc.) and click *Apply* and *Finish.*
    ix. Click *execute* from the main relax window to start the hetNOE analysis.
    x. Once the calculations are finished, open the specified results directory. The hetNOE output file will be stored as **noe.700.out.** Change the extension of the file from .out to .txt and import it into Excel to check the residue-specific hetNOE values and to plot graphs of hetNOE values against the residue number for the respective data.
      ***Alternatives:*** Use Graphpad Prism to plot graphs.
    xi. Determine the average hetNOE value by taking the median and standard deviation of the hetNOE values obtained for the residues.

2. Measurement on the NMR instrument with magnetic field strength B_2_ (for e.g., 21.1 T with a ^1^H-frequency 900 MHz)
  a. Repeat all the steps outlined under 1. at the magnetic field strength B_2_.

### Step 2: Determination of longitudinal relaxation rates (*R*_1_) at two magnetic fields

**Timing: 1–2 weeks**

Longitudinal relaxation (*T*_1_), also called spin-lattice relaxation, is the relaxation process by which the net magnetization returns to the equilibrium as a function of time. Determination of *T*_1_ relaxation involves quantifying the recovery rate of net magnetization aligned with the applied magnetic field (B_0_) using time-dependent recovery of intensity (Kay et al., 1989). Longitudinal relaxation rate (*R*_1_), the simple inverse of the *T*_1_, is most-commonly determined by using the inversion recovery experiment (Figure 2B). This experiment involves the application of 180° pulse to perturb the magnetization from *z* to -*z*-axis, followed by a variable delay (to allow magnetization to relax to *z*), and subsequent 90 ° pulse to flip the magnetization from *z*-axis to the *xy* plane for the detection.

3. Measurement of longitudinal (*R*_1_) relaxation on the NMR instrument with magnetic field strength B_1_
  a. Data acquisition
    i. Create a new dataset and experiment.
    ii. Load the Bruker pulse sequence for the *R*_1_ relaxation measurement (e.g., *hsqct1etf3gpsi*).
    iii. Perform further steps as described in 1a. iii–xii.
    iv. Adjust the relaxation delay (*d7*) to the desired relaxation delay for e.g., 0.4 s.
    v. Type zg to start the acquisition.
    vi. Perform the above-mentioned steps to record experiments with effective relaxation delays such as 0.6, 0.8, 1.2, 1.6, 2.0 and 2.4 s.
  b. Data processing
    i. After all the experiments are finished, process the first spectrum (delay 0.4 s) using standard processing parameters and the 2D Fourier transformation command xfb. Note that this spectrum will have peaks with negative intensity. Determine the phase correction values and process with the 2D Fourier transformation command xfb. Note the intensity scaling factor for this spectrum by typing nc_proc in the topspin command line.
    ii. Process all the remaining spectra using the same processing parameters as the first spectrum. Type in the command line xfb nc_proc x, where x intensity scaling factor (integer value) for the first spectrum.
    iii. Before opening spectra in the NMRFAM-Sparky, convert all the Topspin processed Bruker files into the .ucsf files using the *bruk2ucsf* macro from the NMRFAM-Sparky.
    iv. Open NMRFAM-Sparky. Click on *File*, *Open* and load all the processed .ucsf spectra files with variable delays from the *R*_1_ relaxation experiment.
    v. Transfer resonance assignments from the BMRB (see before you begin, 6. BMRB assignment transfer).
    vi. Save the project by clicking on *File* followed by *Project* and *Save As…* and provide a suitable File name for the project.
    vii. Type lt to open the peak list. To display the column displaying data height for each peak, click on the *Options* and check the *Data height* box. Click *Apply* and then *Close.*
    viii. Click *save* to save the list.
    ix. Perform the above-mentioned steps for each spectrum.
  c. Data analysis using relax GUI
    i. Open *relax-GUI* and start new *R*_1_ *relaxation analysis* by clicking on *File*, *New analysis* and *R*_1_ *relaxation* to fit the obtained data heights for each residue *via* NMRFAM-Sparky to determine the *R*_1_ relaxation rates*.*
    ii. Enter *NMR frequency*, *Results directory path* and load *spin system* as outlined in the hetNOE analysis.
    iii. In the main relax window, click on the *Add* button under the *Spectra list* option to open the *Peak intensity loading wizard* and load the data file names by loading sequentially peak list files (associated with each relaxation delay such as 0.4, 0.6, 0.8, 1.2, 1.6, 2.0 and 2.4 i.e., in total seven) exported from the NMRFAM-Sparky followed by specifying the relevant spectrum ID Strings (1, 2, 3 etc.). Click *Apply* and *Next.*
    iv. The next step is required for the error analysis. The *Baseplane RMSD* using the noise obtained from NMRFAM-Sparky for each spectrum took a very long time for the calculations as well as resulted in the program crash. To bypass this problem, set the *Baseplane RMSD* value of 10 for each spectrum and use a statistical bootstrapping scheme to determine the associated errors for the *R*_1_ relaxation rates (see following steps x and xi).
    v. In the following step assign the relaxation time (in seconds) such as 0.4, 0.6, 0.8, 1.2, 1.6, 2.0 and 2.4 to the respective spectrum ID string and click *Apply* and *Finish.*
    vi. Set *Exponential curve model* to the inversion recovery. It is the three-parameter fit model which employs the parameters such as $R_{x}$, *I*_0_ and *I*_∞_ and the following equation
      $$I_{\left(t\right)}=I_{\infty }-I_{0}*{\text{e}}^{\left(-R_{x}*t\right)}$$
      This equation takes into account that the magnetization proceeds from the negative value at *-I*_0_ to finally relax as a positive *I*_*∞*_ value.
    vii. Click execute from the main relax window to start the *R*_1_ *relaxation analysis.*
    viii. Once the calculations are finished, open the specified results directory. The *R*_1_ relaxation output file will be stored as **r1.700.out.** Change the extension of the file from .out to .txt and import it into Excel to check the residue-specific *R*_1_ relaxation rates and to plot residue-wise graphs of *R*_1_ relaxation rates.
    ix. Determine the average *R*_1_ relaxation rate by taking the median and standard deviation for the rates obtained for all the residues.
    x. To employ a statistical bootstrapping scheme for determining the associated uncertainty, iterate above mentioned (i–viii) steps (at least) three times by alternatively omitting two peak lists (i.e., use five peak lists instead of seven peak lists) for determining the *R*_1_ relaxation rate.
    xi. Determine the standard deviation for the *R*_1_ relaxation rates from these four datasets (one original + three from bootstrapping) for each residue to calculate the associated uncertainty.

4. Measurement of longitudinal (*R*_1_) relaxation on the NMR instrument with magnetic field strength B_2_
  a. Repeat all the steps outlined under 3.

***Alternatives:*** An alternative to the inversion recovery method is the saturation recovery method, which allows quicker *T*_1_ measurement, though inversion recovery is generally preferred for better sensitivity and its robustness with respect to the radiofrequency inhomogeneity as well pulse imperfections. For high molecular weight proteins TROSY-based experiments (Zhu et al., 2000), which offer better sensitivity than the related HSQC-based experiments, would be an ideal choice. We recommend the optimized pulse sequences from (Lakomek et al., 2012) which are implemented in the Bruker pulse sequence library (*trnoeetf3gpsi3d.3*, *trt1etf3gpsitc3d.3*, *trtretf3gpsitc3d.3*).

### Step 3: Determination of transverse relaxation rates (*R*_2_) at two magnetic fields

**Timing: 1–2 weeks**

Transverse relaxation (*T*_2_), also called as spin-spin relaxation, is the relaxation process for the decay of excited net magnetization perpendicular to the applied magnetic field (B_0_) with respect to time (Kay et al., 1989). One of the most-commonly used procedures to determine the *T*_2_ is to measure transverse relaxation in the rotating frame i.e., *T*_1__ρ_. Transverse relaxation rate in the rotating frame (*R*_1__ρ_), the simple inverse of the *T*_1__ρ_, is determined by measuring the decay of a signal influenced by the spin-lock conditions to generate a rotating magnetic field near to the *xy* plane perpendicular to the static magnetic field (Figure 2C) (Massi et al., 2004). In this experiment, spins are excited by the application of a 90° pulse followed by a variable spin-lock period (Δ). A series of experiments with varying spin-lock periods are recorded to quantify the *R*_1__ρ_ rate. The *R*_1__ρ_ rate is then used to derive the *R*_2_ relaxation rate.

5. Measurement of transverse (*R*_1__ρ_) relaxation on the NMR instrument with magnetic field strength B_1_
  a. Data acquisition
    i. Create a new dataset and experiment.
    ii. Load the Bruker pulse sequence for the transverse (*R*_1__ρ_) relaxation measurement (e.g., *hsqctretf3gpsi*).
    iii. Perform further steps as described in 1a. iii–xii.
    iv. Adjust the relaxation delay (*d31*) to the desired relaxation delay (e.g., 0 ms) to record the first dataset.
    v. Start acquisition by typing zg in the command line.
    vi. Perform the above-mentioned steps to record experiments with effective relaxation delays such as 15, 25, 35, 45, 55, and 65 ms.
  b. Data processing
    i. Determine the phase correction values for the first spectrum (delay 0 ms) and process it using standard processing parameters and the 2D Fourier transformation command xfb.
    ii. Perform further data processing steps same as *R*_1_ relaxation data processing (as described in 3b. ii–ix).
  c. Data analysis using relax GUI
    i. To determine the *R*_1__ρ_ relaxation rates from the obtained data heights for each residue with variable delay via NMRFAM-Sparky, open relax-GUI and start new *R*_2_ relaxation analysis by clicking on File, New analysis and *R*_2_ relaxation.
    ii. Perform steps to load NMR frequency, Results directory path, spin system and Spectra list as outlined in the R_1_ relaxation analysis (see 3c. ii–iv).
    iii. In the following step assign the relaxation time (in seconds) such as 0, 0.015, 0.025, 0.035, 0.045, 0.055 and 0.065 to the respective spectrum ID string and click Apply and Finish.
    iv. Set Exponential curve model to the Two parameter exponential fit model. In this model, the magnetization begins at *I*_0_ decaying towards zero. It employs parameters such as $R_{x}$ and *I*_0_ and uses the following equation
      $$I_{\left(\text{t}\right)}=I_{0}\text{∗}{\text{e}}^{\left(-R_{x}\text{∗t}\right)}$$
    v. Next click *Execute* button from the main relax window to start the analysis.
    vi. The output file for the *R*_1__ρ_ relaxation rates will be stored as **r2.700.out** in the results directory. Import it into i.e., Microsoft Excel after changing file extension to .txt. Subsequently analyze and plot *R*_1__ρ_ relaxation rates with respect to the residue numbers. Determine the associated uncertainity using statistical bootstraping scheme (see 3c. x,xi).
    vii. Calculate *R*_2_ relaxation rate for each residue from the *R*_1__ρ_ relaxation rate using the following equation
      $$R_{1\text{ρ}}=R_{1}{\text{ cos}}^{2}\text{θ}+R_{2}{\text{ sin}}^{2}\text{θ,}$$
      where $\text{θ}={\text{tan}}^{-1}\left({\text{ν}}_{1}/\text{Δν}\right)$ and Δν is the offset of the rf field to the resonance (Massi et al., 2004).
    viii. Determine the average *R*_1__ρ_ and *R*_2_ relaxation rates by taking the median and standard deviation for the values obtained for all the residues.
    ix. Perform error propagation to determine the errors for *R*_2_ relaxation rates by using *R*_1_ and *R*_1ρ_ rates and associated errors.

6. Measurement of transverse (*R*_1__ρ_) relaxation on the NMR instrument with magnetic field strength B_2_
  a. Repeat all the steps outlined under 5.

***Alternatives:*** An alternative to the *T*_1__ρ_ measurements is the direct measurement of *T*_2_ involving magnetization decay. However, *T*_2_ measurements are often complicated by the phase inhomogeneity and hence, generally considered unreliable. *T*_1__ρ_ measurements are more suitable for determining *R*_2_ relaxation rates in the low-microsecond timescale. A very good alternative to determine *R*_2_ relaxation rates higher than this time window, is to employ TRACT (TROSY for rotational correlation times)-based approach (Lee et al., 2006).

### Step 4: Validation using theoretical (HYDRONMR computed) relaxation rates

This step is performed to crosscheck the experimentally determined ^15^N relaxation rates against the theoretically predicted relaxation rates using HYDRONMR program (Garcia de la Torre et al., 2000). Refer to the program manual for the complete description of the program and example files: http://leonardo.inf.um.es/macromol/programs/hydronmr/hydronmr.htm

**Timing: 1–2 h**

7. HYDRONMR Calculation
  a. Create a directory for the HYDRONMR calculation and copy the protein *pdb* file in the directory.
  b. Create an HYDRONMR input file *hydronmr.dat.*
  c. Prepare an input file *hydronmr.dat* file by providing information about parameters such as the name of the molecule, name for the output file, name of the input PDB file, AER value (3.3 Å), which is the average value of the atomic element radius for most proteins which ranges in between 2.8 – 3.3 Å (Bernado et al., 2002), temperature (298 K), Magnetic fields (16.4, 18.8 T etc.) and interatomic distance for the N-H bond (1.02 Å), Chemical shift anisotropy (−173 ppm etc.).
  d. The values for *NSIG*, *SIGMIN* and *SIGMAX*, *ETA*, *IFLAG*, *Gyromagnetic ratio*, *no. of values* of the magnetic field can be left unaltered.
  e. Upon successful completion of the run, the output will be stored in the same directory.
  f. Open the output file containing main results file with extension *.res* in a text editor.
  g. Analyze the results and subsequently open it in Microsoft Excel or Graphpad Prism to plot graphs with respect to the residue numbers.

***Note:*** HYDRONMR output file (.res) also contains the information about theoretically calculated diffusion properties such as translational diffusion coefficient, rotational diffusion coefficient, rotational diffusion anisotropy and mean rotational correlation time.

### Step 5: Determination of rotational correlation time and diffusion tensor.

**Timing: 4–8 h**

The rotational correlation time (τ_c_) is the time taken by a protein molecule to rotate by one radian in solution thus reflecting its overall size (Gaspari and Perczel, 2010). Longitudinal (*T*_1_) and transverse (*T*_2_) ^15^N relaxation times derived from the experimentally derived relaxation rates such as (*T*_1_ = 1/*R*_1_) and (*T*_2_ = 1/*R*_2_) are used to determine the rotational correlation time (τ_c_) (Kay et al., 1989; Rossi et al., 2010) by using the following empirical equation:

$$\tau_{c}\approx \frac{1}{4\pi \nu_{N}}\sqrt{6\frac{T_{1}}{T_{2}}-7}$$

where ν_N_ is the resonance frequency of ^15^N in Hz.

The rotational diffusion tensor is determined using TENSOR2 (Dosset et al., 2000) program which uses the experimentally determined backbone relaxation data and protein three-dimensional structural coordinates as an input. The complete description of the program is found at

http://rmni.iqfr.csic.es/HTML-manuals/TENSORV2_DOC/Practice.html

8. Determination of rotational correlation time
  a. Open Excel sheet and import residue-wise *R*_1_ and *R*_2_ relaxation rates and associated uncertainties determined from step 2 and step 3.
  b. Take the simple inverse of *R*_1_ and *R*_2_ relaxation rates to determine *T*_1_ and *T*_2_ relaxation times, respectively.
  c. Calculate τ_c_ for each residue from *T*_1_ and *T*_2_ relaxation times using the above equation for τ_c_ as a function of the *T*_1_ and *T*_2_ relaxation times.
  d. Determine the average τ_c_ by taking the median and standard deviation for the values obtained for all the residues.
  e. Perform error propagation to determine the errors for τ_c_ by using *R*_1_ and *R*_2_ rates and associated errors.
  f. Repeat the above steps to determine the τ_c_ from the second magnetic field strength.

***Alternatives:*** The TENSOR2 program can also be used to determine the rotational correlation time.

9. Determination of rotational diffusion tensor
  a. Create a directory and launch TENSOR2 program in the terminal with *tensor2.*
  b. Click on *File*, *Open data file* and load the input ^15^N relaxation data file containing *R*_1_, R_2_, hetNOE relaxation rates along with the respective uncertainty values.
  c. Next, in the TENSOR2 main window, click on *File*, *Open PDB file* and load the pdb file for the protein of interest.
  d. Click on *Visualization*, *Definition to view*, select or deselect the residues used for the calculations.
  e. Click on *Setup, Spectro* to enter the spectrometer frequency.
    ***Optional:*** Go to the lower menu and select *Isotropy*, *Fit* to determine the τ_c_. Click on *Visualization*, *R1/R2 Tc* to check the results.
  f. From the lower menu, Select *Anisotropy* and click on *Fit* to calculate the diffusion tensor. This calculates the principal components of the diffusion tensor for axially symmetric (*D*_||_ and *D*_⊥_) and fully anisotropic (*D*_xx_, *D*_yy_, *D*_zz_) models.
  g. Set *Nb Cycle* to 500 and click on *Start Monte-Carlo* to determine the associated uncertainty.
  h. Text files *resaniso.0* and *resaniso.1* contain the results of fits with Monte-Carlo simulations, respectively.
    ***Alternatives:*** relaxGUI can be used instead of TENSOR2 to determine diffusion parameters.

### Step 6: Model-free analysis

**Timing: 1–2 weeks**

Model-free analysis allows to extract physically meaningful dynamic motional analyses in a quantitative form on multiple timescales from the experimental steady state NOE, *R*_1_ and *R*_2_ relaxation data using the formalism initially developed by (Lipari and Szabo, 1982a, b). The following text contains steps to perform model-free analysis using the fully automated d’Auvergne protocol in the relaxGUI mode. For the detailed description refer to the relax manual (https://www.nmr-relax.com/manual/).

10. Model-free calculation using relaxGUI
  a. Open relaxGUI and start new model-free analysis by clicking on the File, new analysis, Model-free analysis button followed by the *Next* and the *Start* buttons.
  b. Start new Steady-state NOE analysis by clicking on *File*, *New analysis*, *Steady-state NOE.*
  c. First define the directory path for the results by clicking on the *change* button in front of the *Results directory.*
  d. Next define the spin systems by clicking on the *spin editor* button. Spin editor window will be launched. Load the spin system for protein backbone spins @N and @H as described above.
  e. Next step is to load the ^15^N relaxation data by clicking on the *Add* button in the *relaxation data* list.
  f. Add relaxation data from the hetNOE, *R*_1_ and *R*_2_ analyses using *relaxation data loading wizard.* The steps include specifying the relaxation data identification string (for e.g., R1_700, R2_700 etc.), the relaxation data type (R1, R2 etc.), and the frequency in Hertz and the relaxation data file. Specify the file format using *Free format file settings* option. Click *Apply* after loading each data type (in total 6 data sets) and click on *Next* option to load the data.
  g. Next load the metadata, which contains information regarding the measurements of *peak intensities*, *temperature control* and *calibration method.* If there was no temperature control and calibration method use, select the option for *no control* applied.
  h. In the next step click on the *dipolar relaxation* option in the main relax window to define the Dipole-dipole interaction. Click on the *Next* button and confirm the preloaded average distance of 1.02 Å. Click *Next* and then the *Finish* option to finalize the setup.
  i. Next, define the Chemical Shift Anisotropy by clicking the *OK* option to the preloaded an averaged CSA value of −172 ppm.
  j. Further, confirm the spin isotopes selections by opening X isotope and H isotope wizards and simply clicking on the *OK* options.
  k. Next, return to the main relax window and leave the settings for the local τ_m_, Model-free models, Grid search increments, Monte Carlo simulation number, Maximum interactions as well as Protocol mode as it is and click on the *Execute* relax option to start the model-free analysis.
11. Data interpretation
  a. Relax output will be stored in the specified directory with separate folders for each model name.
  b. Open the final folder which will contain the results files such as *S2.out*, *rex.out* and *te.out* which contains the output for *S*^2^, *R*_ex_ and τ_e_ respectively.
  c. Change the extension of these files from .out to .txt and import it into Microsoft Excel to check the residue-specific values and plot graphs (using Micrososft Excel or Graphpad Prism) against the residue number for the respective data.

***Alternatives:*** FAST Modelfree (Cole and Loria, 2003) and TENSOR2 (Dosset et al., 2000) can be used instead of relaxGUI to perform Model-free analysis. A major caveat for TENSOR2 is that it uses data from only a single magnetic field for the model-free analysis which could lead to inaccurate findings.

## Expected outcomes

This protocol was used to characterize the functional plasticity of the bacterial Tudor domains but with small changes might also be applied to other structurally homologous protein domains for obtaining a thorough understanding of the functional variations associated with the protein backbone dynamics. The described experimental procedure to determine ^15^N{^1^H}-NOE, longitudinal (*R*_1_) and transverse (*R*_2_) relaxation rates combined with the model-free analysis provide a useful understanding of the motions displayed by the protein NH groups on the fast to slow timescale regimes, which can be applied on the uncharacterized protein to understand its motional spectrum or can be used as a compare and contrast approach as described in this study.

**Figure 3:**
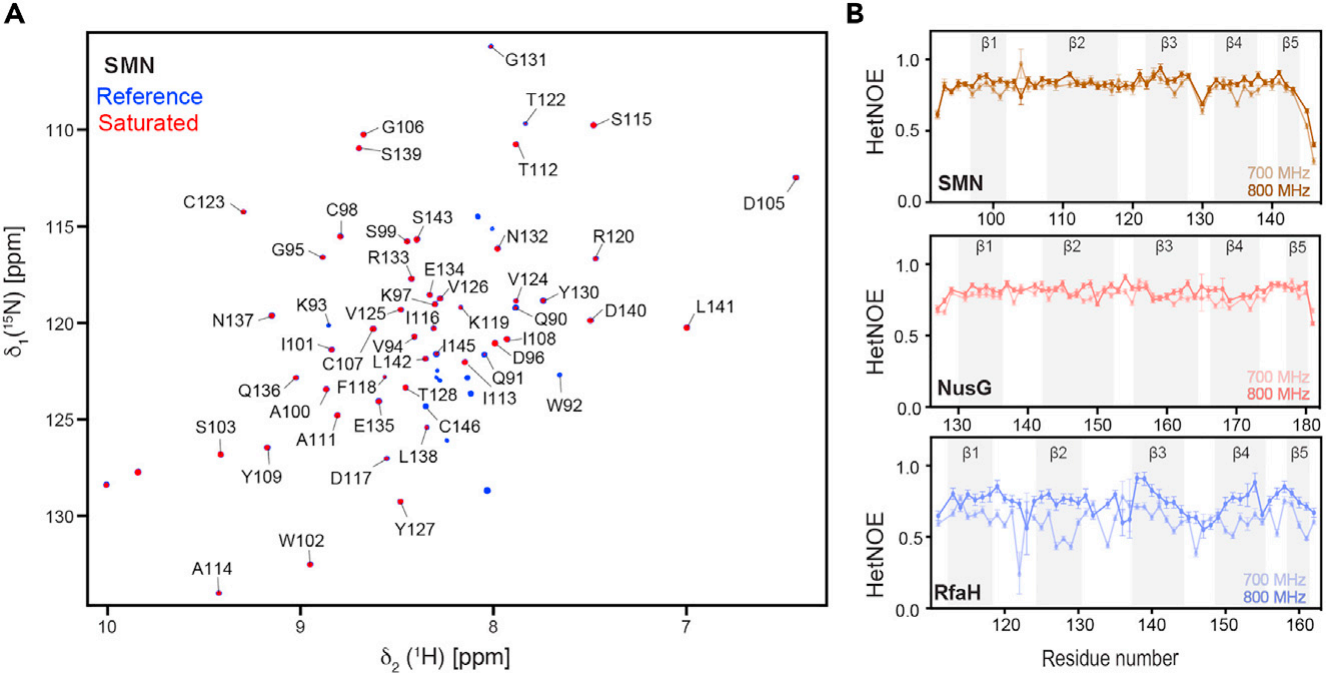
Comparative hetNOE analysis (A) Representative example of the ^15^N{^1^H}–heteronuclear NOE experimental data shown by the overlay of reference and saturated spectrum for the SMN Tudor protein. Single-letter residue name and the respective residue number are used to annotate the assignments and the positions of the backbone amide resonances. (B) The steady-state ^15^N{^1^H}–heteronuclear NOE data at 700 and 800 MHz magnetic fields plotted against the residue numbers for the SMN, NusG and RfaH Tudor domains. Secondary structure elements are indicated by the grey background. Adapted from (Kawale and Burmann, 2021) with permission from Elsevier. Error margins were obtained via the relaxGUI program using estimated root-mean-square base plane noise of the spectra.

**Figure 4:**
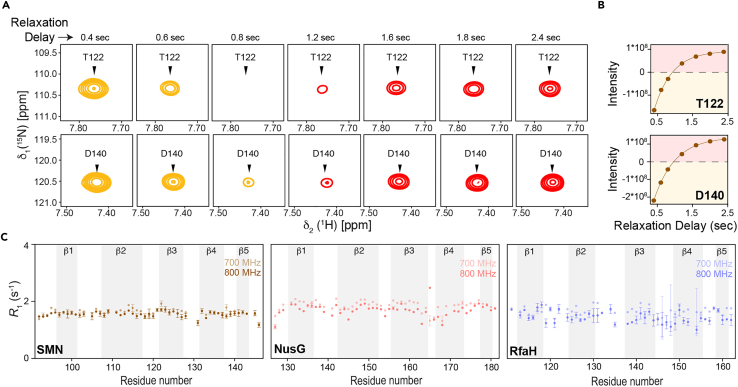
Comparison of longitudinal relaxation rates (*R*_1_) (A) Representative examples of the longitudinal relaxation (*R*_1_) measurement data (inversion recovery method) depicted by the intensity changes. (B) The associated relaxation data profiles for the residues T122 and D140 of the SMN Tudor protein at 700 MHz. Negative and positive intensity peaks are shown in orange and red, respectively. (C) Residue-wise plots of the ^15^N longitudinal relaxation rates for the Tudor domains under study. Secondary structure elements are indicated by the grey background. Adapted from (Kawale and Burmann, 2021) with permission from Elsevier. Error margins for the *R*_1_ relaxation rates were determined using the statistical bootstrapping scheme.

**Figure 5:**
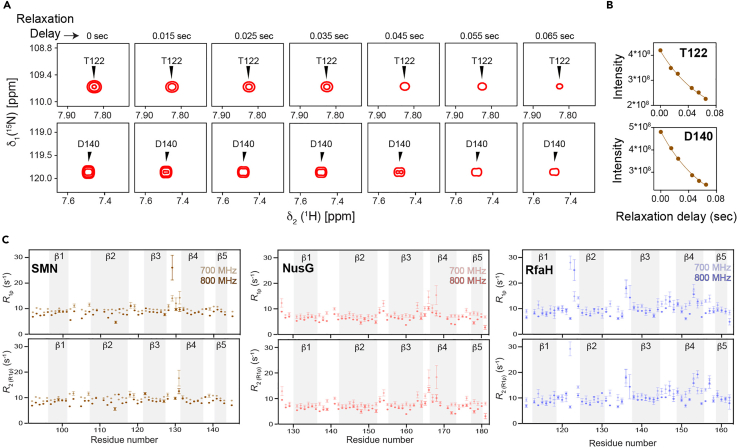
Comparison of transverse relaxation rates (*R*_2_) (A) Examples of the Transverse relaxation (*R*_2_) measurement data represented by the intensity decay. (B) The associated relaxation data profiles for the residues T122 and D140 of the SMN Tudor protein at 700 MHz. (C) ^15^N transverse relaxation rates plotted against residue numbers for the Tudor domains under study. Secondary structure elements are indicated by the grey background. Adapted from (Kawale and Burmann, 2021) with permission from Elsevier. Error margins for the *R*_1ρ_ relaxation rates were determined using the statistical bootstrapping scheme. *R*_2_ error margins were determined using the error propagation from the *R*_1_ and *R*_1ρ_ error margins.

**Figure 6:**
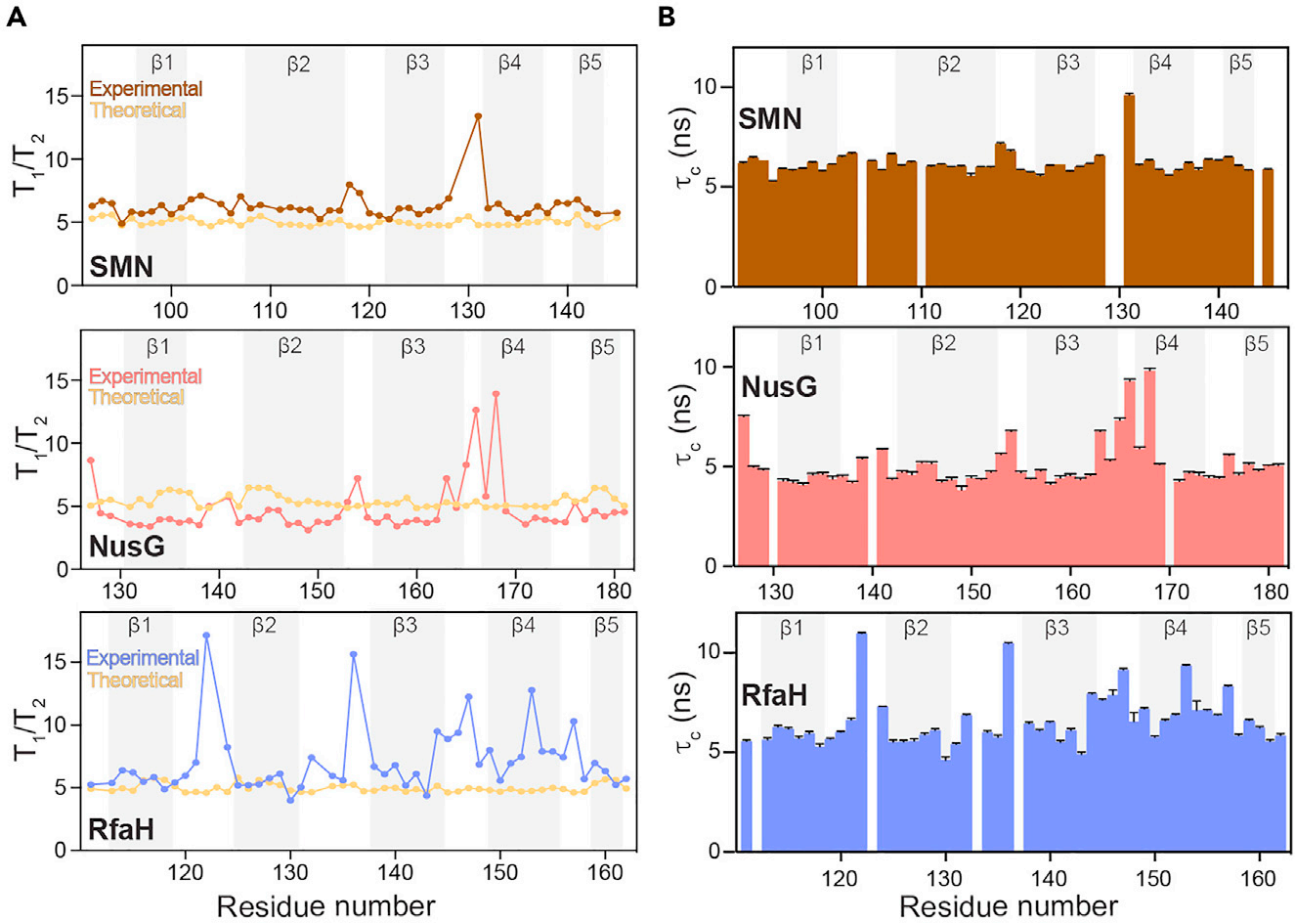
Validation against HYDRONMR computed values and rotational correlation times of Tudor domains (A) Comparison between the theoretical (HYDRONMR predicted) versus the experimental values of the *T*_1_/*T*_2_ ratio. (B) Backbone relaxation NMR data based rotational correlation time (τ_c_) plotted against the residue numbers of the Tudor domains with secondary structure elements highlighted by the grey background. Adapted from (Kawale and Burmann, 2021) with permission from Elsevier. Error margins for the rotational correlation times were determined using the error propagation from the *R*_1_ and *R*_1ρ_ error margins.

Our comprehensive backbone relaxation data analysis revealed a drastically different relaxation behavior for the RfaH Tudor domain contrasting the relaxation properties of the SMN and NusG Tudor domains. The presence of extended motions on the fast timescale (ps–ns) was evident by the fluctuating hetNOE values (Figure 3) as well as *R*_1_ rates (Figure 4) across the RfaH Tudor domain. In contrast, we observed nearly planar hetNOE and *R*_1_ relaxation profiles for the SMN and NusG Tudor domains. Similarly, we also observed enhanced *R*_2_ relaxation rates predominantly for the loop residues of the RfaH Tudor domain in comparison to the SMN and NusG Tudor domain (Figure 5). The analysis of experimental versus HYDRONMR computed relaxation rates and rotation correlation times for each Tudor domain is presented in Figure 6.

**Figure 7:**
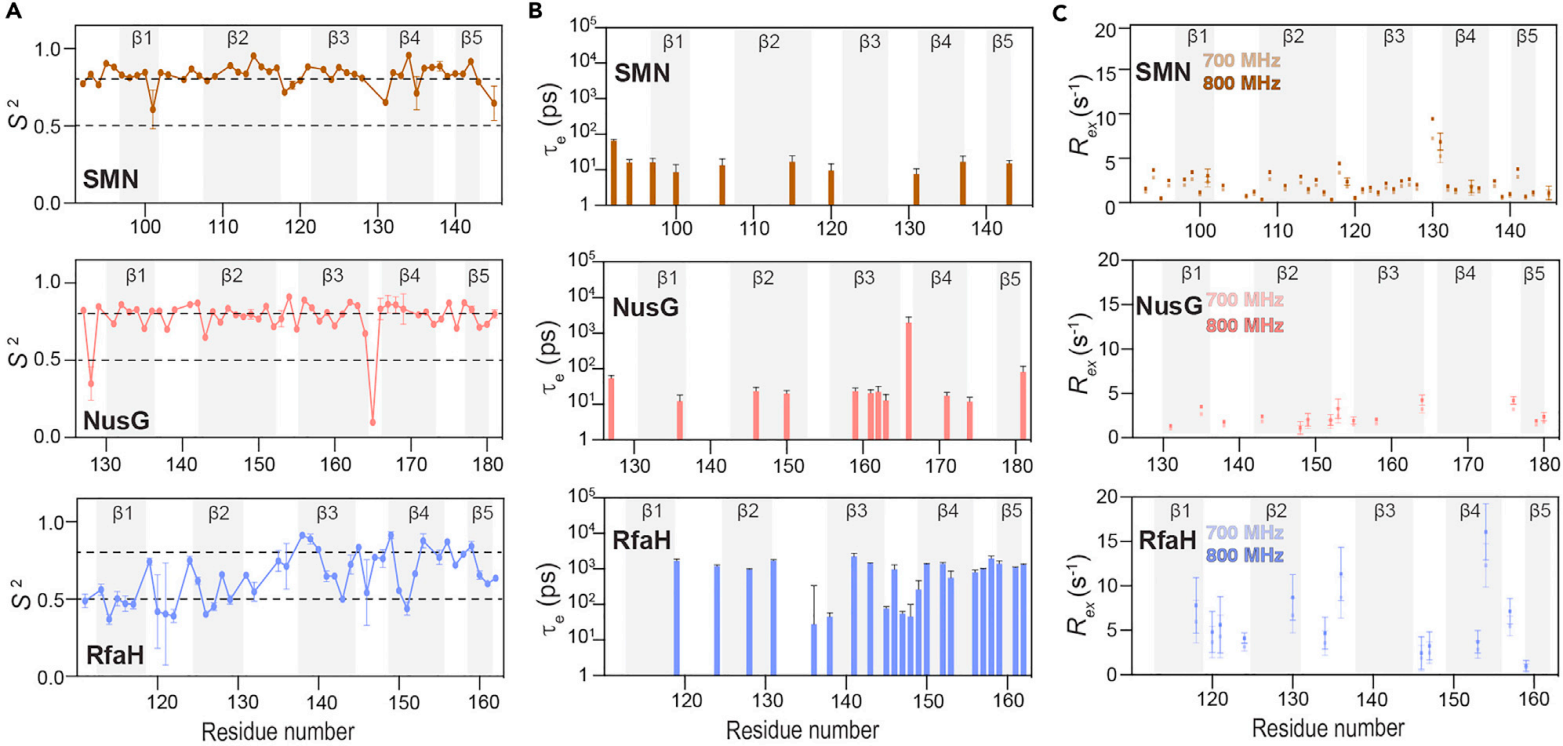
Results from the Model-free analysis Residue-wise plots of (A) the generalized order parameters (*S*^2^), (B) the calculated τ_e_ values representing internal motions faster than the rotational correlation time, and (C) conformational exchange (*R*_ex_) values for each Tudor domain under study. The broken lines in panel A indicate an *S*^2^ of 0.5 and 0.8 distinguishing highly flexible (*S*^2^ < 0.5) and structurally rigid (*S*^2^ > 0.8) regions. Adapted from (Kawale and Burmann, 2021) with permission from Elsevier. Error margins were obtained from the relaxGUI program.

The differential relaxation behavior of the RfaH Tudor domain was further ascertained by the Model-free analysis revealing a strong fluctuation (ranging from 0.37 to 0.91) in the generalized order parameter, (*S*^2^), revealing the presence of extensive amplitudes of motions (Figure 7). The strong presence of τ_e_ terms in the carboxy-terminal part of the protein reflected the motions on the ps–ns timescale whereas the presence and the stronger magnitudes of *R*_ex_ terms corroborated the presence of slow timescale motions predominantly by the loop residues. In contrast, the rigidity of the SMN and NusG Tudor proteins is reflected by the higher *S*^2^ values along with the fewer τ_e_ and *R*_ex_ terms.

Thus, our in-depth backbone relaxation data analysis discerns the crucial differences between the dynamic properties of the structurally similar Tudor domains corroborating the conformational plasticity and the metamorphic behavior of the RfaH Tudor.

## Quantification and statistical analysis

NMR data were analyzed using NMRFAM-sparky (Lee et al., 2015). Error margins for the hetNOE data were obtained from the spectral noise as outlined in detail above. For the *R*_1_ and *R*_1__ρ_ rates a statistical bootstrapping scheme was used to determine the error margin of the relaxation rates as in more detail described in the respective sections. These error margins were propagated for the determination of the margins of the *R*_2_ rates as outlined above. The used programs, relaxGUI (Bieri et al., 2011) and Tensor2 (Dosset et al., 2000), use a Monte-Carlo based scheme directly embedded in the respective software; details can be obtained from the respective software documentation as indicated above.

## Limitations

This protocol is applicable to a wide array of protein domains as well as protein-ligand complexes ranging from small to medium-size proteins. But there are limitations regarding the protein size. Higher molecular weight proteins display strong signal overlaps and in addition tumble more slowly in solution and thus, leads to the line broadening of the resonances and reduced signal sensitivity. Although deuteration and TROSY-based relaxation experiments (Lakomek et al., 2012; Zhu et al., 2000) could help in increasing the signal sensitivity, protein size pose limitation to the application of this protocol.

Unavailability of high field spectrometers with two magnetic fields and long data measurement time could also limit the application of this protocol. The symmetry requirement approximation in the Model-free approach, also limits the use of this protocol to the proteins with definite protein shapes, and hence for intrinsically disorders proteins application of this protocol is not valid and the reader is referred to more specialized approaches such as i.e., developed by the Blackledge group (Adamski et al., 2019; Milles et al., 2018).

## Troubleshooting

### Problem 1

Sample deteriorates over time.

### Potential solution

Use 0.02% sodium azide solution to prevent bacterial or fungal growth in the samples. When not measuring data, remove the sample from the NMR tube and flash freeze it in liquid nitrogen and subsequently store it at −80°C. If possible, use lower protein concentrations 0.1–0.2 mM for the measurement to avoid concentration dependent aggregation which might increase over time. To prevent degradation of the sample, add a few μl of protease inhibitor cocktail solution into the sample. Typically, ∼5 μL of one cOmplete, EDTA-free Protease Inhibitor Cocktail tablet (Roche) dissolved in the respective buffer is sufficient. If the sample requires DTT, add fresh DTT from the stock as DTT is prone to degradation over time. If not limited with the material, aliquot the sample in several batches and store at −80°C and use a fresh sample for each data measurement to ensure consistent data for unstable samples.

### Problem 2

Low signal to noise for the NMR signals.

### Potential solution

Increase the sample concentration used for the measurement unless protein is aggregation-prone or limited by the material availability otherwise increase the number of scans.

### Problem 3

*R*_1_ and *R*_2_ relaxation data curves are not ideal.

### Potential solution

Increase the range of delays used for the data measurement to obtain ideal data curves for *R*_1_ and *R*_2_ relaxation data. A good approximation of a suitable time-range is that the signal intensity at the last measurement point is at least ∼I0e to ensure good sampling of the relaxation data curve.

### Problem 4

Calculations are computationally too demanding and time consuming.

### Potential solution

Use NMRbox (Maciejewski et al., 2017), which provides all the required software mentioned in the protocol on cloud based virtual machines, offering faster calculations for computationally demanding steps. In addition, using NMRbox has the advantage, that the required software does not need to be installed locally and eventually needs to be compiled for the local computer used.

### Problem 5

Overlapping peaks complicates the estimation of individual peak height or volume.

### Potential solution

Use peak integration programs, e.g., PINT (Ahlner et al., 2013), specifically designed for this purpose. If the residues causing overlapping peaks are not crucial for the analysis (for e.g., terminal residues) exclude them from the analysis.

### Problem 6

Limited NMR spectrometer time and/or limited sample stability preventing the full dynamical analysis required.

### Potential solution

In recent years developments of nonuniform sampling (NUS) methods have found broad applicability in the NMR community as a time-saving method in NMR spectroscopy (Jaravine et al., 2006; Mobli and Hoch, 2014). However, quantitative analysis of such spectra has been complicated by the non-linearity of the signal intensities preventing its broad usage (Linnet and Teilum, 2016; Stetz and Wand, 2016). Nevertheless, latest developments employing accordion spectroscopy (Bodenhausen and Ernst, 1982) with nonuniform sampling providing comparable data quality as conventional approaches offers a promising route under time-limited circumstances (Carlstrom et al., 2019).

## Resource availability

### Lead contact

Further information and requests for resources and reagents should be directed to and will be fulfilled by the lead contact, Björn M. Burmann (bjorn.marcus.burmann@gu.se).

### Materials availability

This study did not create new unique reagents.

## Data Availability

The NMR data used for the relaxation analysis have been tabulated and are available on Mendeley data: https://doi.org/10.17632/3thwpmz88s.1
